# Locally existing endothelial cells and pericytes in ovarian stroma, but not bone marrow-derived vascular progenitor cells, play a central role in neovascularization during follicular development in mice

**DOI:** 10.1186/1757-2215-7-10

**Published:** 2014-01-21

**Authors:** Fumie Kizuka-Shibuya, Nobuko Tokuda, Kiyoshi Takagi, Yasuhiro Adachi, Lifa Lee, Isao Tamura, Ryo Maekawa, Hiroshi Tamura, Takashi Suzuki, Yuji Owada, Norihiro Sugino

**Affiliations:** 1 Department of Obstetrics and Gynecology, Yamaguchi University Graduate School of Medicine, Minamikogushi 1-1-1, Ube 755-8505, Japan; 2 Department of Organ Anatomy, Yamaguchi University Graduate School of Medicine, Minamikogushi 1-1-1, Ube 755-8505, Japan; 3 Department of Pathology, Tohoku University School of Medicine, 2-1 Seiryo-machi, Aoba-ku, Sendai 980-8575, Japan

**Keywords:** Angiogenesis, Vasculogenesis, Parabiosis, Follicle growth, Pericyte, Vascular endothelial cell

## Abstract

**Background:**

Neovascularization is necessary for follicular growth. Vascularization is first observed in preantral follicles, and thereafter the vasculature markedly increases in follicles undergoing development. Neovascularization includes angiogenesis and vasculogenesis. Vasculogenesis is the formation of new blood vessels by bone marrow-derived endothelial progenitor cells. It is unclear whether vasculogenesis occurs during follicular growth. Blood vessels must be mature to be functional blood vessels. Mature blood vessels are characterized by the recruitment of pericytes. However, it is unclear where pericytes come from and whether they contribute to neovascularization in the follicle during follicular growth. In this study, we investigated whether bone marrow-derived progenitor cells that differentiate into vascular endothelial cells or pericytes contribute to neovascularization during follicular growth.

**Methods:**

A parabiosis model was used in this study. Six-week-old wild-type and transgenic female mice expressing green fluorescent protein (GFP) were conjoined between the lateral abdominal regions to create a shared circulatory system. After 6 weeks, the ovaries were obtained and immunostained for CD31/CD34 (a vascular endothelial cell marker), platelet-derived growth factor receptor-β (PDGFR-β) (a pericyte marker), and GFP (a bone marrow-derived cell marker).

**Results:**

Cells that were positive for CD34 and PDGFR-β were observed in the stroma adjacent to the primary or early preantral follicles and in the theca cell layer of the follicles from the late preantral stage to the preovulatory stage. CD31/CD34 and GFP double-positive cells were observed in the theca cell layer of the follicle from the antral stage to the preovulatory stage while the number of double-positive cells in the preovulatory follicles did not increase. PDGFR-β and GFP double-positive cells were observed in the theca cell layer of the preovulatory follicle but not in the smaller follicle.

**Conclusions:**

Locally existing endothelial cells and pericytes in the stroma play a central role in the neovascularization during follicular growth, while bone marrow-derived endothelial cells and pericytes partially contribute to this process.

## Background

Angiogenesis is required for follicular growth during the early follicular developmental stage [1]. Injection of vascular endothelial growth factor (VEGF), a principal angiogenic factor, into the ovarian bursa stimulates the growth of preantral follicles [2]. Inhibition of angiogenesis by VEGF inhibitors prevents the growth of antral follicles, leading to an increased number of atretic follicles and a lack of ovulatory follicles [3]. Suppression of angiogenesis in preantral and early-antral follicles causes follicular atresia at these follicular developmental stages [4]. Furthermore, vascular development also plays a crucial role in the selection and maturation of the dominant follicle destined to ovulate during the late follicular developmental stage [1]. In fact, inhibition of angiogenesis in the late follicular phase interferes with the final stage of follicular development and delays ovulation [5,6].

Primordial follicles and early-preantral follicles do not have their own individual vascular supply, but instead rely on blood vessels in the surrounding stroma [7]. Soon after the antrum has appeared in the follicle, the follicle acquires a vascular sheath in the theca cell layer [7,8]. Vascularization is first observed in follicles containing 4 granulosa cell layers (preantral follicles) [9], and thereafter the vasculature markedly increases in follicles undergoing development from the preantral stage to the antral stage. Regarding the onset of vascularization of the theca cell layer, it has been thought that endothelial cells are recruited to the thecal layer from the blood vessels in the adjacent stroma. Interestingly, we recently found that bone marrow-derived vascular progenitor cells contribute to neovascularization during corpus luteum formation, suggesting the involvement of vasculogenesis in corpus luteum formation [10]. Neovascularization includes angiogenesis and vasculogenesis. Angiogenesis is the development of new blood vessels by endothelial cell proliferation and outgrowth from pre-existing blood vessels. Vasculogenesis refers to new blood vessel formation by bone marrow-derived vascular progenitor cells. Vasculogenesis is a characteristic phenomenon in embryogenesis, but it has been reported to play a role in neovascularization in variety of organs, including those in the adult body [11,12]. Given this new understanding of adult neovascularization, it is also possible that vasculogenesis is responsible for neovascularization during the follicular growth. Therefore, it is important to investigate whether vasculogenesis occurs during follicular growth.

The vasculature in the follicle delivers oxygen, nutrients, hormones, and bioactive substances for follicular growth and final selection of the dominant follicle. Therefore, blood vessels in the follicle need to stabilize and mature to be functional. Maturation of blood vessels is characterized by the recruitment of pericytes. Pericytes serve as structural components of blood vessels that maintain vascular integrity [13,14]. Recently, we reported that pericytes are present in the ovary and contribute to neovascularization during the corpus luteum formation in mice [10] although the presence of pericytes in the rodent ovary has been controversial [15]. However, it is unclear how the pericyte distribution changes during follicular growth, where pericytes come from, and whether pericytes contribute to neovascularization in the follicle during follicular growth. One possibility is that progenitor cells of the pericytes come from the bone marrow and play a role in neovascularization during follicular growth.

In this study, we investigated whether bone marrow-derived progenitor cells that differentiate into vascular endothelial cells or pericytes contribute to neovascularization during follicular growth.

## Materials and methods

### Animals

C57BL/6NCrSlc mice were purchased from Japan SLC, Inc., Hamamatsu, Japan. Green fluorescent protein (GFP)-expressing mice (C57BL/6-Tg(CAG-EGFP)), were kindly supplied by Masaru Okabe (Genome Research Center, Osaka University, Osaka, Japan). The mice were housed at 24°C under controlled conditions (lights on from 0500 to 1900 h) under specific pathogen-free conditions and were fed by standard chow and maintained. All experimental protocols were reviewed by the Ethics Committee for Animal Experimentation of Yamaguchi University School of Medicine and carried out according to the Guidelines for Animal Experimentation of the Yamaguchi University School of Medicine and under the Law and Notification requirements of the Japanese Government.

### Parabiosis model

Bone marrow transplantation (BMT) model has generally been used to detect bone marrow-derived vascular progenitor cells. In this model, bone marrow-derived vascular progenitor cells are tracked by transplanting bone marrow cells from GFP-transgenic mice into the recipient mice whose immune systems were compromised by irradiation. The irradiation damages the ovaries [10], which makes it difficult to examine physiological development of follicles in the BMT model. In addition, when the mice are treated with gonadotropins, the damaged basement membrane of the preovulatory follicle allows vascular endothelial cells to enter the granulosa cell layer before ovulatory LH surge [10]. Furthermore, low dose irradiation is reported to stimulate vasculogenesis in various organs [16,17]. For these reasons, we used the parabiosis model, in which two mice are conjoined subcutaneously without irradiation and share a common circulation, in order to determine whether endogenous circulating vascular progenitor cells contribute to neovascularization in the follicle during the follicular development.

**Figure 1:**
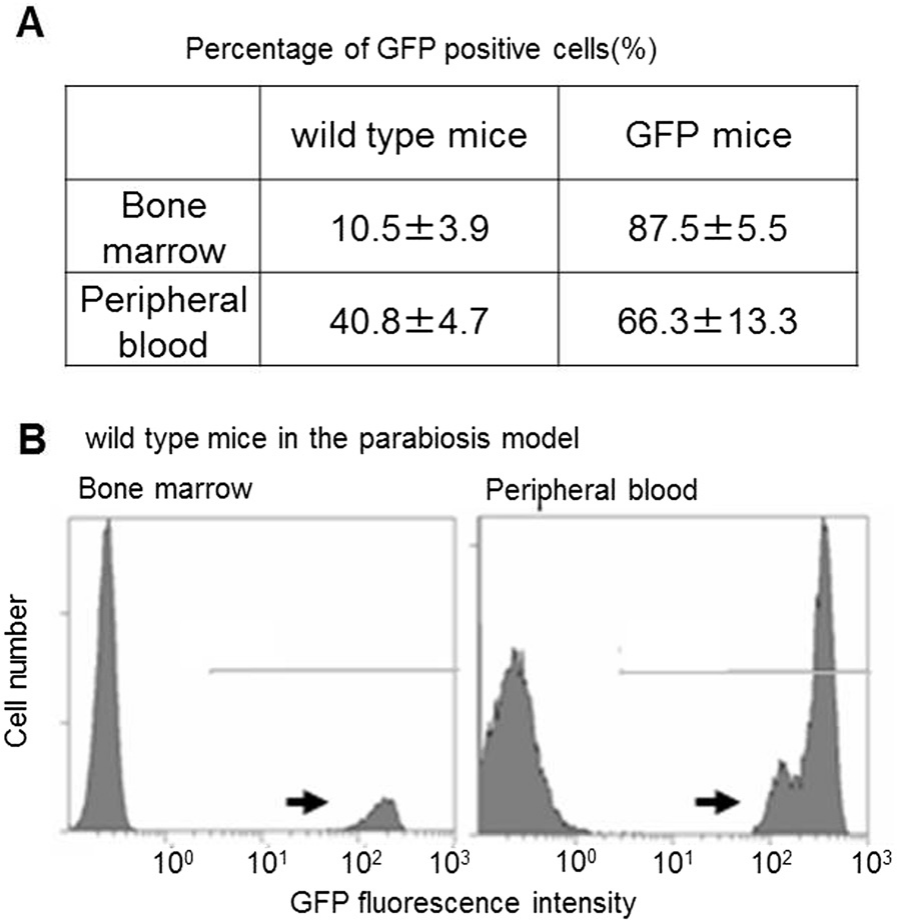
**Proportion of cells expressing GFP in the bone marrow and in the peripheral blood in the parabiosis model.** To evaluate the successes of the parabiosis model, the percentage of the cells expressing GFP was analyzed in the bone marrow and in the peripheral blood by flow cytometry 6 weeks after parabiosis. Values are mean +/− SD of three animals **(A)**. Representative cases of flow cytometry in the parabiosis model are shown **(B)**. Arrows indicate cell population expressing GFP.

Pairs of sex- and weight-matched, 6-week-old wild-type and GFP-transgenic mice were parabiosed as described previously [18,19]. In brief, the mice were anesthetized by intraperitoneal injection each of 50 μg/kg pentobarbital (Kyoritsu Chemical Industries, Ltd., Tokyo, Japan) diluted in saline. The corresponding lateral aspects of each mouse were shaved and sterilized with 70% ethanol. A longitudinal skin incision was made from the olecranon to the knee joint of each mouse, after dissection of the subcutaneous connective tissue along the incision, the corresponding skin edges were approximated symmetrically, slightly converted, and joined with a 3–0 monocryl suture. To validate whether parabiosis succeeded, the percentage of the cells expressing GFP was checked in bone marrow and in peripheral blood by flow cytometry after 6 weeks. In the parabiosis model, cross-circulation was established between the two mice for both humoral factors and cellular blood components. Partner-derived cells were observed rapidly and stably not only in peripheral blood but also in bone marrow. Blood and bone marrow were taken from the right ventricle and the femurs, respectively, under diethylether anesthesia. The red blood cells and bone marrow cells were hemolysed with Immunoprep Reagent System (Beckman Coulter, Inc., Fullerton, CA, USA). Cells were suspended in 2% BSA/PBS at a concentration of 1× 10^6^ cells/ml followed by incubation with propidium iodide (Sigma-Aldrich Co., St. Louis, MO, USA). Flow cytometry was performed using an FC500 cytometer (Beckman Coulter, CA, USA). Bone marrow cells (10.5%) and peripheral blood cells (40.8%) were replaced by GFP positive cells (Figure 1).

### Immunohistochemistry

Wild type mice in the parabiosis model were transcardially perfused with 4% paraformaldehyde in phosphate buffer under inhalation anesthesia using Forane (isoflurane; Abbott Laboratories, IL, USA). For fluorescence-based immunohistochemistry, the ovaries were removed and immersed in phosphate buffer containing 30% sucrose and then embedded in OCT compound (Sakura Finetek Co., Ltd, Tokyo, Japan). Sections (10 μm) were prepared on a cryostat (CM 1850, Leica Microsystems Inc, IL, USA). The fluorescence-based immunohistochemistry was performed as described previously [20]. The primary antibodies used in this study were rat anti-mouse CD31 (an endothelial cell marker; 550274; Becton, Dickinson and company, Franklin Lakes, NJ, USA, dilution 1/50) and rabbit anti-platelet-derived growth factor receptor β antibodies, a pericyte marker (PDGFR-β, dilution 1/2000). Anti-PDGFR-β antibodies were kindly supplied by Dr. William B. Stallcup (Burnham Institute, La Jolla, CA, USA), and the specificity of the rabbit anti-PDGFR-β antibodies is well established [10,21]. Anti-goat IgG-Alexa594 (Invitrogen Corp., Carlsbad, CA, USA, dilution 1/100) were used as the secondary antibodies. After counterstaining with 4′,6-Diamidino-2-phenylindole (DAPI, 0.5 μg/ml, Invitrogen) the sections were covered with Fluoromount (Diagnostic BioSystems, CA, USA) and observed using a confocal laser microscope (LSM510 META; Carl Zeiss, Oberkochen, Germany). For negative controls, sections were incubated with normal goat serum instead of primary antibodies.

For enzyme-based immunohistochemistry, ovaries were embedded in paraffin. Paraffin-embedded ovaries were sectioned at 4 μm and the sections were deparaffinized in xylene and rehydrated in a graded series of ethanol. The enzyme-based immunohistochemistry was performed as described previously [13]. The primary antibodies used in the enzyme-based immunohistochemistry were rat anti-mouse CD34 (an endothelial cell marker; HM1015; Hycult Biotechnology, Uden, the Netherlands, dilution 1/50) and rabbit anti-PDGFR-β antibodies (dilution 1/1000). When CD34 antibodies were used, the tissues were microwaved at 95°C for 20 min in 0.01 M citrate containing 0.1% Tween 20 epitope retrieval buffer (pH 6.0). The sections were subsequently visualized using ABC (avidin-biotinylated peroxidase complex) system (Vector Laboratories, Inc., Burlingame, CA, USA) with 3,3′-diaminobenzidine tetrahydrochloride (3,3′-DAB), and were counterstained with hematoxylin. For negative controls, sections were incubated with normal goat serum instead of primary antibodies.

Double immunohistochemical staining for GFP and CD34 was performed as described previously with some modifications [22]. Briefly, deparaffinized sections were incubated with the rabbit anti-GFP antibody (ab290; Abcam plc., Cambridge, UK, dilution 1/2000) and subsequently visualized with 3,3′-DAB. After the sections were placed in boiling citric acid buffer (pH 6.0) for 20 min, the slides were incubated with the rat anti-mouse CD34 antibody (dilution 1/50), and visualized by True Blue (KPL, Inc., Gaithersburg, MD, USA). Methyl green was used for counterstaining the nuclei. For double immunostaining for GFP and PDGFR-β, sections were incubated with the rabbit anti-PDGFR-β antibody (dilution 1/200), visualized with DAB solution, reacted with anti-GFP antibody (dilution 1/5000), and then visualized by Vector Blue (Vector Laboratories, Burlingame, CA, USA). No counterstaining was performed in this double immunostaining.

For immunohistochemistry, six to twelve ovaries obtained from three to six parabiosis models were used, and immunostaining was evaluated on three to four tissue sections in each ovary. Developmental stages of follicles were defined by the following criteria: 1) primordial follicle; oocytes surrounded by a single flat layer of follicular somatic cells, 2) primary follicle; oocytes surrounded by a single layer of cuboidal granulosa cells, 3) early preantral follicle; 2–4 layers of granulosa cells, 4) late preantral follicle; more than 4 layers of granulosa cells and no antrum, 5) early antral follicle; each follicle containing an antrum, diameter < 200 μm, 6) late antral follicle; follicle containing an antrum, diameter > 200 μm, and 7) preovulatory follicle [23].

## Results

**Figure 2:**
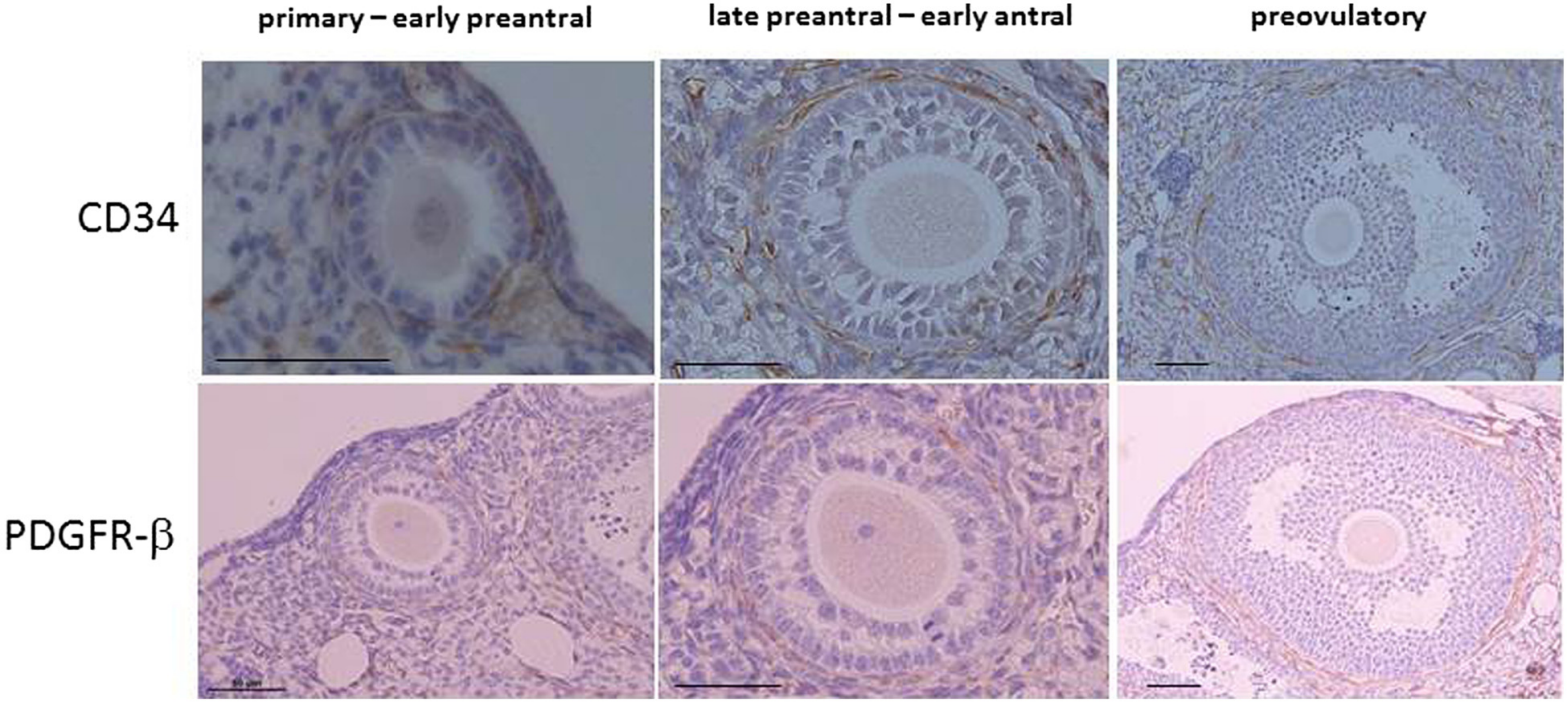
**Immunohistochemical detection of CD34 (a vascular endothelial cell marker) and PDGFR-β (a pericyte marker) in the parabiosis model.** Six to twelve ovaries were obtained from three to six wild-type mice in their natural estrous cycles. Immunostaining was evaluated on three to four tissue sections in each ovary in each developmental stage of the follicles. The developmental stages are defined in Materials and Methods. Scale bars; 50 μm.

CD34-positive cells and PDGFR-β-positive cells were observed in the stroma adjacent to the primary or early preantral follicle. They were also observed in the theca cell layer of the follicles from the late preantral stage to the preovulatory stage by enzyme-based immunohistochemistry (Figure 2).

**Figure 3:**
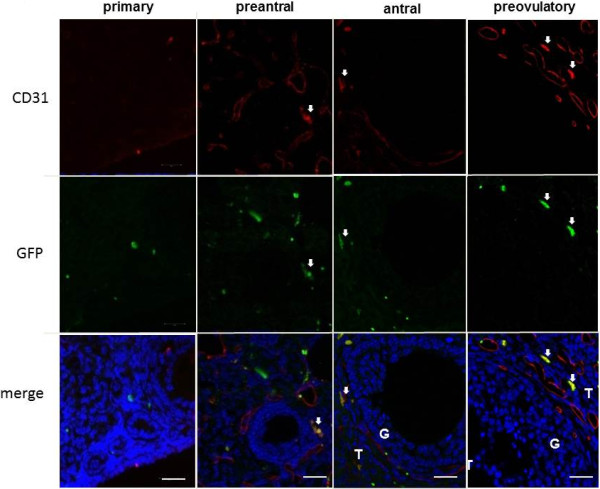
**Fluorescence-based immunodetection of CD31 (a vascular endothelial cell marker) in the parabiosis model.** Six to twelve ovaries were obtained from three to six wild-type mice in their natural estrous cycle. Immunostaining was evaluated on three to four tissue sections in each ovary in each developmental stage of the follicles. The developmental stages are defined in Materials and Methods. Arrows indicate CD31 and GFP double-positive cells. T: theca cell layer, G: granulosa cell layer. Scale bars; 20 μm.

**Figure 4:**
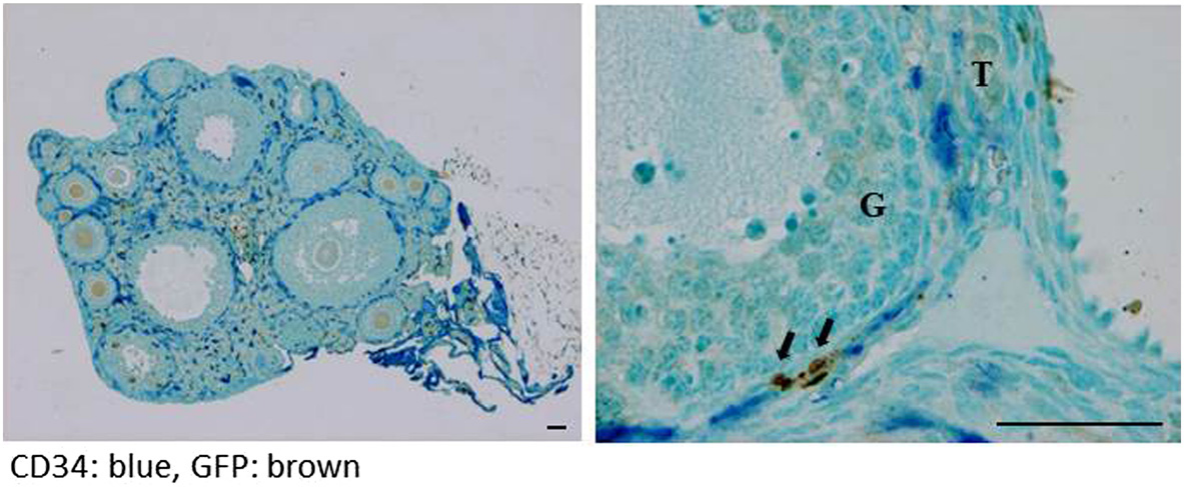
**Double immunostaining for CD34 (a vascular endothelial cell marker) and GFP (a bone marrow derived-cell marker) in the parabiosis model.** Blue shows CD34, and brown shows GFP. Double-positive cells are indicated by arrows. T: theca cell layer, G: granulosa cell layer. Scale bars; 50 μm.

The fluorescence-based immunohistochemistry showed that CD31 and GFP double-positive cells were present in the stroma adjacent to the preantral follicle and in the theca cell layer of the follicle from the antral stage to the preovulatory stage, but not around the primary follicle (Figure 3). Double immunohistochemical staining for GFP and CD34 by enzyme-based immunohistochemistry also showed the presence of double-positive cells in the theca cell layer of the preovulatory follicle (Figure 4, right). These findings indicate the presence of bone marrow-derived vascular endothelial cells around the preantral follicle and in the theca cell layer of the antral and preovulatory follicles. Although the number of double-positive cells for CD31/CD34 and GFP was too small to count (Figure 4, left), the proportion of GFP-positive cells in CD31/CD34-positive cells was estimated to be less than 3%. This indicates that only a few bone marrow-derived endothelial cells were incorporated into the blood vessels by the mechanism of vasculogenesis.

**Figure 5:**
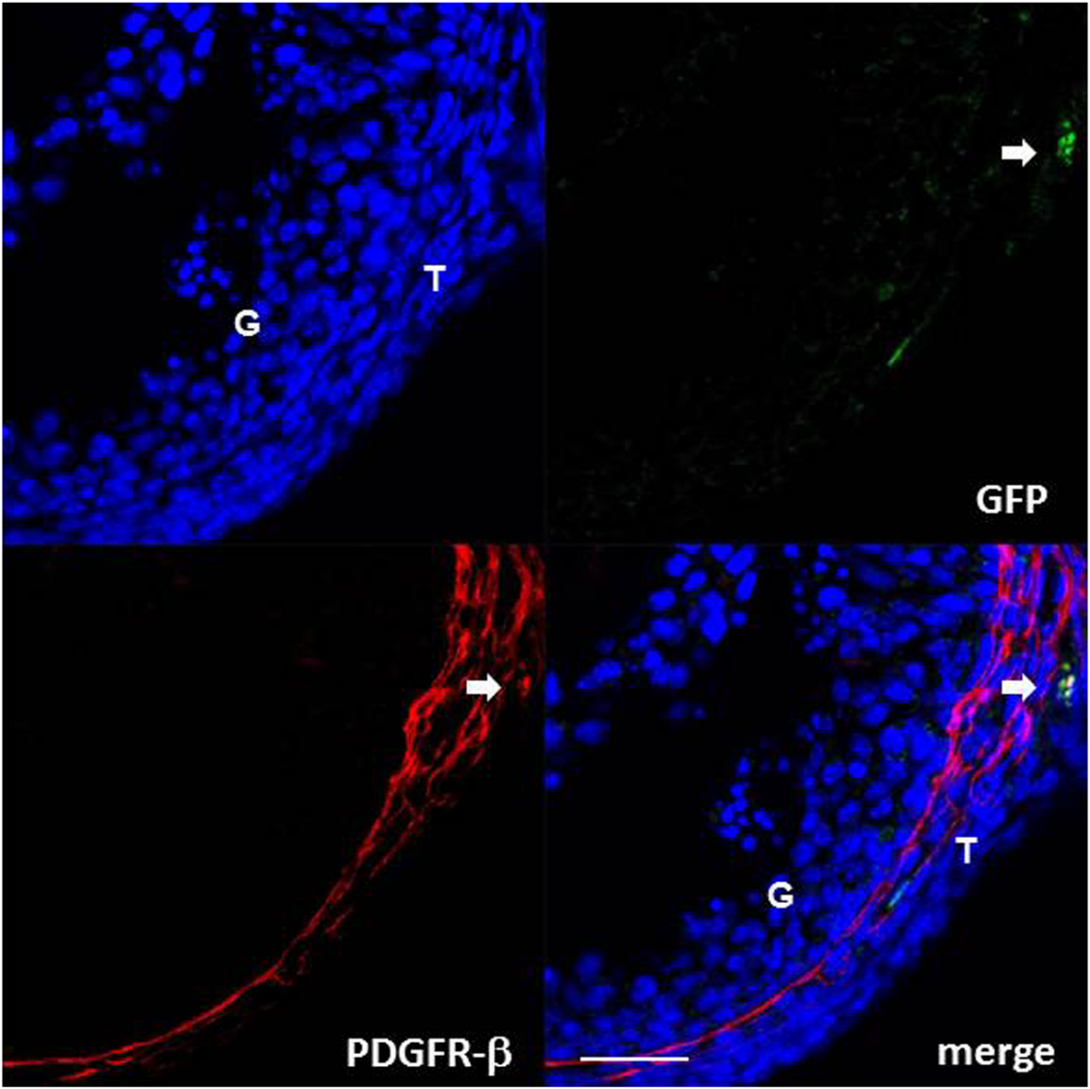
**Fluorescence-based immunodetection of PDGFR-β (a pericyte marker) in the parabiosis model.** PDGFR-β is shown as red. GFP is shown as autofluorescence green. Arrows indicate PDGFR-β and GFP double-positive cells. Immunostaining was evaluated on three to four tissue sections in each ovary in each developmental stage of the follicles. The developmental stages are defined in Materials and Methods. T: theca cell layer, G: granulosa cell layer. Scale bars; 20 μm.

**Figure 6:**
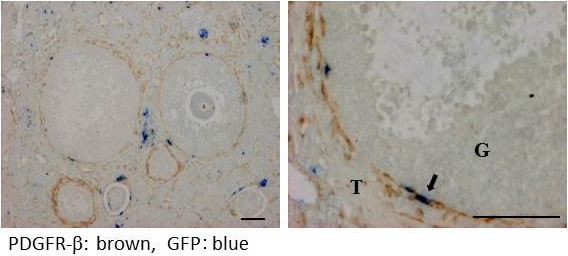
**Double immunostaining for PDGFR-β (a pericyte marker) and GFP (a bone marrow derived-cell marker) in the parabiosis model.** Brown shows PDGFR-β and blue shows GFP. Double-positive cells are indicated by arrows. T: theca cell layer, G: granulosa cell layer. Scale bars; 50 μm.

PDGFR-β and GFP double positive cells were observed in the theca cell layer of the preovulatory follicle, but not in the small antral follicle, as shown by both fluorescence-based (Figure 5) and enzyme-based immunohistochemistry (Figure 6, right). These findings indicate the presence of bone marrow-derived pericytes in the theca cell layer of the preovulatory follicle, but not in the small antral follicles. The double-positive cells for PDGFR-β and GFP were too few to count (Figure 6, left), but were estimated to count for less than 3%.

## Discussion

Angiogenesis plays important roles in the growth of the recruited cohort of small antral follicles in the early follicular developmental stage and subsequent selection of a dominant follicle during the late follicular developmental stage. Vascular endothelial cells form the blood vessels in the follicle, but origin of these cells is unclear. Here, we used a parabiosis model to show the presence of vascular progenitor cells that differentiate into vascular endothelial cells in the follicle of the various developmental stages. This suggests that vasculogenesis at least partly contributes to neovascularization during follicular growth. Vasculogenesis also has roles in neovascularization during corpus luteum formation [10], uterine endometrial growth [24], and wound healing [25,26]. However, in the present study, only a few bone marrow-derived endothelial progenitor cells were observed to be incorporated into the blood vessel of the follicle by vasculogenesis. Furthermore, the numbers of these cells in the follicles did not increase from the early developmental stage to the preovulatory stage, suggesting that bone marrow-derived endothelial cells do not proliferate during follicular growth to form the vascular network of the follicles. Therefore, it appears that locally existing vascular endothelial cells around the follicle in the stroma, rather than bone marrow-derived endothelial cells, are recruited to the theca cell layer and proliferate for neovascularization during the follicular growth. Interestingly, bone marrow-derived endothelial progenitor cells indirectly contribute to neovascularization by stimulating proliferation or invasion of preexisting vascular endothelial cells [27]. Much attention has been given to the involvement of paracrine signals secreted by endothelial progenitor cells in neovascularization, because endothelial progenitor cells produce various angiogenic factors including growth factors and cytokines [25,27]. Presently, it is thought that the main role of bone marrow-derived endothelial progenitor cells in neovascularization is to stimulate proliferation or invasion of preexisting vascular endothelial cells [25,27] rather than to directly constitute blood vessels by the mechanism of vasculogenesis [11,12].

Pericytes contribute to blood vessel stabilization with neovascularization in the ovary [10,13,14,28,29]. Our results show that pericytes are present around the preantral follicle and in the theca cell layer of the antral follicles (Figure 2), suggesting that pericytes contribute to neovascularization and blood vessel stabilization during follicular growth. Regarding the origin of pericytes in the follicle, our results also show that bone marrow-derived pericytes are present in the theca cell layer of the preovulatory follicles but not in the smaller antral follicles. The presence of bone marrow-derived pericytes in the theca cell layer of the preovulatory follicle is consistent with our recent report [10]. These findings suggest that locally existing pericytes in the stroma, but not bone marrow-derived pericytes, are recruited to the follicle and proliferate during follicular growth. Although the role of bone marrow derived-pericytes in the preovulatory follicle is unclear, they may indirectly contribute to neovascularization by stimulating proliferation or invasion of preexisting vascular endothelial cells [10,29,30]. Further studies are needed to clarify the mechanism and role underlying the recruitment of bone marrow derived pericytes to the preovulatory follicle.

## Conclusions

Locally existing endothelial cells and pericytes in the stroma play a central role in the neovascularization during follicular growth, while bone marrow-derived endothelial cells and pericytes partially contribute to this process.

## Competing interests

The authors declare that they have no competing interests.

## Authors’ contributions

FKS conceived of the study, contributed to the production of animal models, carried out enzyme-based immunohistochemistry and fluorescence-based immunohistochemistry, and drafted the first manuscript. NT, YA, LL, IT, RM, HT, and YO carried out enzyme-based immunohistochemistry and fluorescence-based immunohistochemistry, and contributed to the production of animal models. KT and TS carried out double immunostaining. NS conceived of the study, directed the research, and drafted the final manuscript. All authors approved the final manuscript.
